# Mechanical Properties and Sulfate Resistance of High Volume Fly Ash Cement Mortars with Air-Cooled Slag as Fine Aggregate and Polypropylene Fibers

**DOI:** 10.3390/ma12030469

**Published:** 2019-02-03

**Authors:** Jun Hyeong Kim, Abdul Qudoos, Sadam Hussain Jakhrani, Atta-ur-Rehman, Jeong Bae Lee, Seong Soo Kim, Jae-Suk Ryou

**Affiliations:** 1 Department of Civil and Environmental Engineering, Hanyang University, Seoul 04763, Korea; bspring@nate.com (J.H.K.); qudoos.engnr@gmail.com (A.Q.); sadamhussain@hanyang.ac.kr (S.H.J.); attabrcian@gmail.com (A.-u.-R.); 2 GFC R&D CO., Ltd., Pocheon-si 11159, Gyenoggi-do, Korea; dlwjdgo@nate.com; 3 Department of Civil Engineering, Daejin University, Pocheon-si 11159, Gyeonggi-do, Korea; sskim@daejin.ac.kr

**Keywords:** fly ash, fiber, air-cooled blast furnace slag aggregate, sulfate attack, mortar

## Abstract

The depletion of natural sand and production of the huge amount of cement in the construction industry are serious threats to the environment, which can be reduced by the utilization of by-products as cement replacement material. In this study, cement was replaced with fly ash up to 45% (by weight). In addition, the natural fine aggregate was replaced with air-cooled blast furnace slag aggregate (here referred to as “slag aggregate”) at a level of 50% and 100% (by weight). Polypropylene fiber was also added, at a dosage of 0.25% of binder weight. Mortar specimens were prepared and analyzed using tests for compressive, flexure, and splitting tensile strength, as well as for microhardness, and ultrasonic pulse velocity. In addition, the specimens were exposed to sulfate solution and investigated for changes in length, mass, and compressive strength. Electron microscopy and X-ray diffraction analysis were performed to examine the microstructure and phase changes of mortar specimens exposed to sulfate solution. The results indicate that mortar specimens made with 50% slag aggregate and 0.25 % fiber showed enhanced mechanical properties. The performance of slag aggregate mortars under sulfate attack was improved significantly.

## 1. Introduction

The production of cement mortar and concrete utilizes huge amounts of natural sand as fine aggregate, leading to a high demand globally for natural sand. Many countries are concerned about the scarcity of natural sand, and the resulting difficulty in meeting construction demands. A decreasing trend for the availability of natural sand for the past 15 years has been reported [1]. To help resolve this issue, there is an increasing effort to utilize waste by-products as fine aggregate for cement mortar and concrete production. The utilization of these by-products not only lowers the cost of cement and concrete manufacturing but also provides reductions in landfill cost, along with energy savings and environmental protection. In addition to this, the utilization of waste by-products has been shown to enhance the microstructure, mechanical, and durability properties of cement composites [2]. 

Blast furnace slag (BFS) is a nonmetallic industrial by-product produced from a molten state in a pig iron blast furnace. Worldwide production of iron slag in 2017 was estimated to be 300–360 million tons [3]. This amount of waste material will be a burden on the environment if it cannot be utilized elsewhere. The classification of BFS depends on the way it is cooled. Air-cooled blast furnace slag is produced by slow cooling under atmospheric conditions, resulting in crystalline mineral formation [4]. In the past two decades, several studies have been conducted to analyze the behavior of concrete incorporating slag aggregates [5,6,7,8]. The results of a study conducted by Yüksel et al. clearly indicated that GBFS used as a sand replacement in an optimal ratio (10–30%) positively affected the durability properties as well as abrasion resistance of concrete [5]. Similarly, self-compacting concrete produced using slag as fine aggregate presented higher compressive strength in the later ages compared to reference concrete [6]. However, autogenous shrinkage and drying shrinkage were negatively affected due to slag inclusion [6]. In another study, results showed that concrete with a higher amount of slag aggregate showed low workability and capillarity water transfer, and increased compressive strength. However, the splitting tensile strength and the modulus of elasticity were similar to those of conventional concrete [7]. Additionally, the strength development of blast furnace slag aggregate mortar was found to be higher, as the reaction continued due to the water present in the voids of the slag aggregate [8].

Fly ash (FA), a by-product produced in coal-fired power plants, is being generated in large quantities that also make it a potential threat to the environment. The utilization of fly ash in cement mortar and concrete has several benefits, including cost reduction, energy efficiency, and a decrease in CO_2_ emissions from cement production. Many researchers have utilized up to 30% fly ash in concrete as cement replacement. The limitation of fly ash replacement in this lower amount is due to the fact that it reduces early strength [9]. On the other hand, incorporation of fly ash in higher volumes provides a benefit, since it lowers the carbon footprint of concrete production by reducing the amount of cement required. High-volume fly ash (HVFA) concrete utilizes 50–70% fly ash as cement replacement. Several studies have been conducted to analyze the properties of high-volume fly ash concrete. Sengul et al. [10] studied the effect of fly ash replacement (up to 70%) on the compressive strength development of concrete. Results showed lower compressive strengths than with plain concrete at early ages (28 days) but higher compressive strength than plain concrete at later ages (56 and 120 days). However, low strength development at the initial ages can be compensated for by incorporating ultra-fine cementitious materials [11,12]. 

Fiber reinforcement of cement composites has been shown to improve ductility, toughness, and tensile strength, and to restrain the progression of cracks. Polypropylene (PP) fiber is a cheap and popular material in the construction industry, and many studies have been conducted to analyze the performance of PP fiber-reinforced cement composites [13,14,15]. Karahan and Atiş [14] studied the durability properties of concrete containing polypropylene fiber. From their results, it is clear that the addition of PP fibers reduced workability and drying shrinkage, while it enhanced compressive strength and freeze-thaw resistance. Research has also shown that porosity, water absorption, and sorptivity coefficient values increased with the increase of fiber content [14]. Similarly, another study found that the presence of polypropylene fiber in concrete reduced the carbonation depth [15]. Therefore, the incorporation of fiber imparts a beneficial effect on the overall performance of concrete. 

Based on a review of the current literature, it is evident that each process: utilizing fly ash in higher amounts, using slag as fine aggregate, and reinforcing cement composites with fiber, has benefits if used individually. However, the combined effects of incorporating these processes have rarely been investigated. Such studies are needed to promote confidence in the utilization of these waste materials in construction projects. Therefore, in the present paper, we aimed to study the efficiency of utilizing slag as fine aggregate in cement mortar, along with a high volume of fly ash as a cement replacement, and polypropylene (PP) fiber reinforcement. Natural sand was replaced with 0%, 50%, and 100% slag fine aggregate, while the fly ash replacement and the fiber addition were fixed as 45% and 0.25% of the total binder weight, respectively. Tests for compressive strength, flexure strength, and splitting tensile strength were conducted. In addition, the mortar specimens were evaluated in terms of sulphate attack, microhardness, ultrasonic pulse velocity, electron microscopy, and X-ray diffraction.

## 2. Materials and Methods

### 2.1. Materials

Ordinary Portland cement (OPC) complying with ASTM C150 [16] was used in this study. Fly ash and silica fume were used as cement replacement materials. The chemical and physical properties (provided by supplier: Sampyo Group, Seoul, Korea) of the OPC, fly ash (FA), and silica fume (SF) are shown in Table 1. Natural sand with a maximum size of 4.75 mm, fineness modulus (F.M) of 2.7, and absorption of 1.05% was used as a fine aggregate. Slag aggregate (Supplied by H company, Gwangyang, Korea) with a maximum size of 4.75 mm, absorption of 3.45%, and Fineness Modulus (F.M) of 3.02 was used as a natural sand replacement. PP fibers with a density of 0.92 g/cm^3^ were used. 

### 2.2. Mix Proportions

The mortar mixes used in this study were given the following acronyms: S0, S50, S100, FS0, FS50, and FS100. Mortar mix types with acronyms beginning with “S” indicate those without fiber addition, while those beginning with “FS” represent fiber-incorporated mortar mixes. The numbers 0, 50, and 100 indicate 0%, 50%, and 100% sand replacement with slag fine aggregate. The fly ash content and the water/binder ratio were fixed at 45% (by weight) of the binder and 0.45, respectively, for all the mortar mixes. Since the use of fly ash in higher amounts compromises the initial strength of cement composites, silica fume was used at a dosage of 5% (by weight) of binder in all the mixes. It has been well established that the addition of silica fume improves early strength development [17,18]. The dosage of silica fume was selected on a trial basis to achieve the desired compressive strength. A fiber dosage of 0.25% of the binder weight was selected for the mortar mixes with fiber inclusion. Fibers were strewed by hand during the mixing process in order to achieve the uniform dispersion of the PP fibers following the procedure mentioned in Reference [15].

### 2.3. Fabrication of Specimens and Testing Methods

Mortar mixes were prepared with a 1:3 ratio of binder to fine aggregate. Mortar cubes (50 × 50 × 50 mm^3^) and prisms (40 × 40 × 160 mm^3^) were prepared in accordance with ASTM C109 [19] and ASTM C348 [20], respectively. Cylindrical specimens (Φ 100 × 200 mm^3^) were prepared for splitting tensile strength test. The mortars were cast in molds, sealed with plastic sheets in order to avoid water evaporation, and kept at room temperature for 24 h. After demolding, the specimens were kept in water at 23 ± 2 °C for curing, until the age of testing was reached. 

Flowability of the mortar mixes was measured in accordance with ASTM C1437 [21]. Compressive strength (ASTM C109 [19]), flexure strength (ASTM C348 [20]), and splitting tensile strength (ASTM C496 [22]) tests were carried out at the ages of 7, 28, 90, and 180 days. For each test, three replicates were used and the average value was reported. Additionally, ultrasonic pulse velocity (UPV) tests were also conducted after 7, 28, 90, and 180 days of water curing in accordance with ASTM C597 [23]. 

The behavior of mortar specimens exposed to sulfate environment was studied by using two types of specimens. For determining length change and mass gain in sulfate solution, mini prisms (10 × 10 × 40 mm^3^) were obtained by cutting the mortar prisms (40 × 40 × 160 mm^3^) using a diamond saw cutter. The mini prisms were then immersed in a sodium sulfate solution. The test method used in this study was similar to those employed by previous researchers [24,25]. Additionally, mortar cubes were used to study the influence of sulfate solution on compressive strength. The procedure for making sulfate solution and measuring length change and mass gains can be found in a previous paper by the authors [25]. It is important to note here that the sulfate solution was maintained at a temperature of 23 ± 2 °C for this study. The mortar cubes immersed in sulfate solution were tested for compressive strength after 28, 90, and 180 days. 

The water-cured and sulfate attack-damaged specimens were analyzed by X-ray diffraction (XRD, RINT D/max 2500, 40 kV, 30 mA, scanning speed 2°/m, wavelength 1.54 Å, Rigaku, Tokyo, Japan) and scanning electron microscopy (SEM, accelerating voltage 0.2–30 kV, Secondary Electron Imaging (SEI) resolution 3.5 nm, probe current 10^−12^ to 10^−5^ A, magnification 10× to 300,000×, model S-3000 N, Hitachi, Tokyo, Japan) equipped with energy dispersive spectroscopy (EDS, detector type SDD Apollo XL, resolution 134.49, take-off angle 35°, accelerating voltage 20 kV). The specimens for SEM and EDS analysis were obtained using the methods described in [26]. The procedure for microhardness testing is discussed in a previous study by the authors [27].

## 3. Results

### 3.1. Flowability

**Figure 1:**
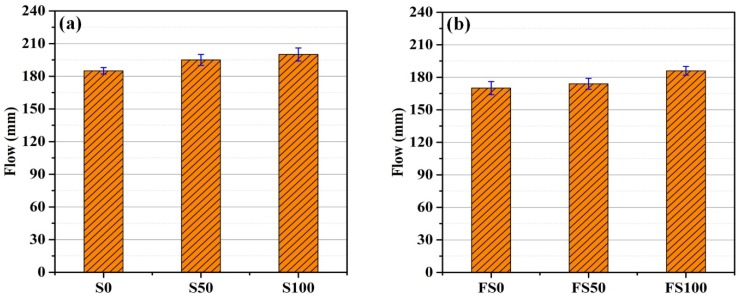
Flow of the mortar mixes (**a**) without fiber and (**b**) with fiber.

Figure 1a depicts the measured flow diameters of each of the non-fiber mortar mixes. It can be seen that the incorporation of slag aggregate increased the flow of both mortar types. The increments in the flow of mortar were 5.4% and 8.1% for S50 and S100 mortar mixes, respectively, in comparison with mortar mixes with only sand. Similar results were observed by Yüksel and Genc [28] in their study, in which workability increased with the addition of slag aggregate. Therefore, the increased workability observed in the present study is due mainly to the release of water that was in the pores of the slag fine aggregate, which increased the water-to-cement ratio.

Figure 1b shows the results of flow table test performed on mortar specimens with PP fibers. The addition of fiber reduced the flow of the mortar mixes. The reduction in flow for FS0, FS50, and FS100 was 8.1%, 10.8%, and 7%, respectively, compared to the corresponding mortar mixes without fiber. It has been reported elsewhere that the inclusion of PP fibers reduced the workability of concrete mixes [14]. The reduction in workability is attributed to the increased adhesion and cohesiveness of concrete mixes that are provided by PP fiber [29].

### 3.2. Mechanical Properties

#### 3.2.1. Mortars without Fiber Addition

**Figure 2:**
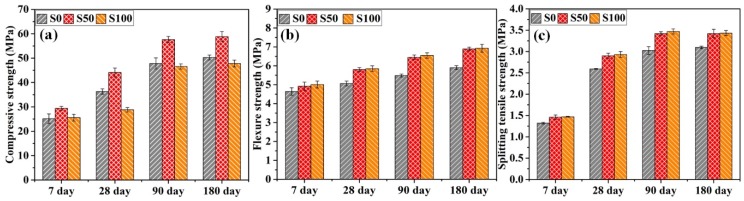
(**a**) Compressive, (**b**) flexure, and (**c**) splitting tensile strengths of the mortar mixes without fiber.

**Figure 3:**
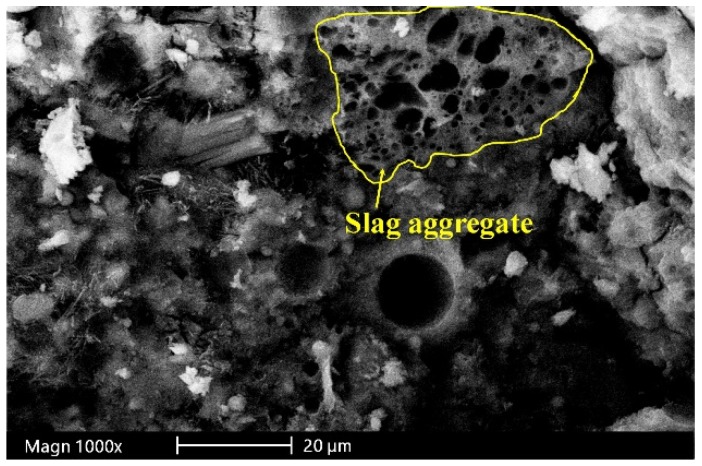
SEM micrograph of slag aggregate in S100 specimen.

Figure 2 shows the results of compressive, flexure, and splitting tensile strengths of the mortar specimens measured at 7, 28, 90, and 180 days. Figure 2a portrays the results of compressive strength test. The compressive strength varied from 25 to 60 MPa. It can be seen that for all curing ages, compressive strength increases significantly with the replacement of natural sand with slag aggregate up to 50%. It suggests the optimal content of the slag aggregate for improvement in compressive strength. On the other hand, compressive strength decreased when the matrix contained 100% slag as fine aggregate. This may be due to the low strength of slag aggregate and the increased porosity of the cement composite made with 100% sand replacement. The porous structure of slag aggregate particle can be seen in Figure 3 (SEM micrograph of S100 specimen). The enhancement in compressive strength with the inclusion of 50% slag was 17.1%, 21.6%, 20.5%, and 17.1%, respectively, at 7, 28, 90, and 180 days of curing. 

Figure 2b presents the results of flexure strength development with respect to time for each mortar mix. Similar to the case of compressive strength, flexure strength increased with the addition of slag aggregate as fine aggregate. However, S100 showed enhancement in flexure strength, while compressive strength for the same mix was reduced. This may be due to the enhanced bonding of slag aggregate with the cement paste. Compared to mortar mix S0, mortar mixes S50 and S100 presented increments, respectively, of 6.1% and 8% at the age of 7 days; 14.6% and 15.6% at the age of 28 days; 17.6% and 19.4% at the age of 90 days; 16.7% and 17.3% at the age of 180 days. 

Splitting tensile strength (STS) tests were conducted on mortar cylinders for each mix at different curing ages, as shown in Figure 2c. The results of the STS tests were in accordance with the flexure strength results. STS increased for specimens with 50% and 100% replacement of sand with slag aggregate. Mortar mixes S50 and S100 showed increases, respectively, of 10.5% and 11.3% at 7 days; 11.9% and 13% at 28 days; 13.1% and 14.7% at 90 days; 10.5% and 10.8% at 180 days, in comparison with the mortar mix S0. 

A study conducted by Escalante-García et al. [8] reported that mortar specimens with GBFS sand showed higher strength than those with silica sand. The strength continued to increase with the curing time, which is attributed to the contribution of GBFS grains in the reaction. The highest strength was reported for mortars with 50% GBFS. The authors also mentioned that the water present in the pores of the slag aggregates was responsible for enhanced strength in the latter ages. In another study conducted by Qasrawi [30], it was mentioned that the angular shape of slag aggregates can be responsible for the higher strength of concrete specimens due to the enhanced bonding of aggregate and cement matrix. The enhanced bonding of the rougher surface aggregate and cement matrix was also reported in a previous study conducted by the authors [27]. Moreover, the study conducted by Topçu & Bilir [31] reported 40% of GBFS as optimal content for improvement in compressive strength and flexure strength. In another study, decreased compressive and flexure strength was reported as the slag replacement exceeded 50% of sand [32].

#### 3.2.2. Mortars with Fiber Addition

**Figure 4:**
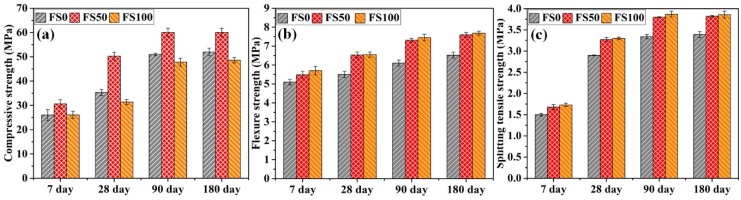
(**a**) Compressive, (**b**) flexure, and (**c**) splitting tensile strengths of the mortar mixes with fiber.

Figure 4 depicts the results of the compressive, flexure and splitting tensile strength tests performed on fiber-incorporated mortar specimens. The results of the compressive strength test are shown in Figure 4a. Compressive strength increased for all the mortar mixes with the incorporation of PP fibers, compared to their respective mixes without fiber inclusion. When compared to corresponding mortar specimens without fiber, FS0, FS50, and FS100 presented increments, respectively, of 3.7%, 4.2%, and 2% at the age of 7 days; 12.9%, 13.9%, and 9% at the age of 28 days; 6.7%, 4.3%, and 2.6% at the age of 90 days; 3.5%, 2.1%, and 1.7% at the age of 180 days. 

The inclusion of PP fiber further increased the flexure strength, as can be seen in Figure 4b. The increments in flexure strength for FS0, FS50, and FS100 were, respectively, 9.9%, 11.4%, 13.8% at the age of 7 days; 8.6%, 12.6%, 12% at the age of 28 days; 11.2%, 13.4%, 13.8% at the age of 90 days; and 10.4%, 10.3%, 10.8% at the age of 180 days, compared to the corresponding mixes without fiber. The addition of fiber also improved the splitting tensile strength of all mortar mixes, as shown in Figure 4c. The increments in STS for FS0, FS50, and FS100 were, respectively, 13.5%, 15.1%, 17.7% at the age of 7 days; 11.9%, 12.8%, 12.6% at the age of 28 days; 10.4%, 11.1%, 11.5% at the age of 90 days; and 9.6%, 11.7%, 12.5%, at the age of 180 days, compared to the corresponding mixes without fiber. Similar results were obtained in a previous study [33], in which compressive and flexure strengths of the mortar specimens were enhanced with the incorporation of PP fibers cured at 20 °C. Additionally, it has been reported that polypropylene fiber–reinforced concrete specimens showed 5.8% and 9.7% increments in compressive strength and splitting tensile strength, respectively, in comparison with those without fiber. This improvement is attributed to the interaction of the fibers with the advancing cracks [34]. However, in another study, the results showed that the incorporation of PP fibers had no significant effect on the compressive strength of lightweight concrete, while flexural strength was enhanced up to 25% [35].

### 3.3. Microhardness Test

**Figure 5:**
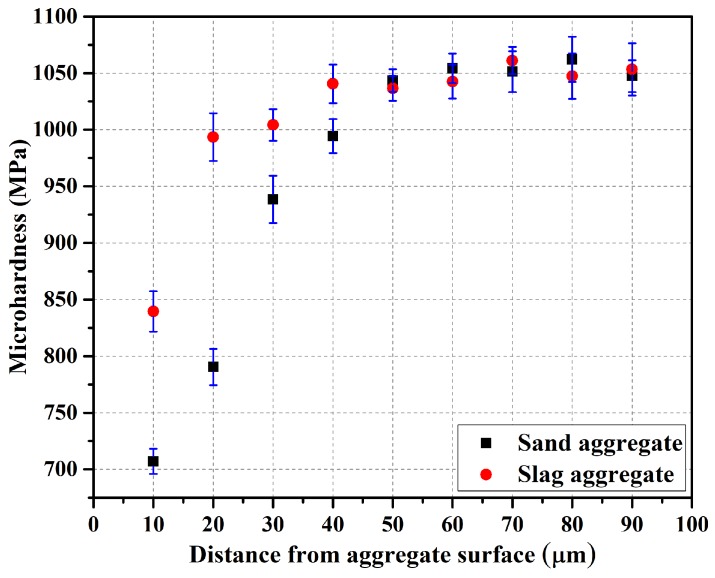
Microhardness profiles along sand and slag aggregates.

Microhardness test was performed on the mortar specimens with slag aggregate and the mortar specimens with sand as a fine aggregate, as shown in Figure 5. The horizontal and vertical axes represent distance in micrometers (µm) from the aggregate surface, and microhardness in megapascals (MPa), respectively. It is obvious from the results that microhardness values varied considerably in the near-surface (i.e., within 50 µm) of the aggregates. The mortar specimens with slag aggregates presented higher microhardness values than those with sand aggregates. An increment of 6.2% in microhardness occurred at a distance of 10 µm from the slag aggregate surface and at 20 µm from the sand aggregate surface. The microhardness values at 20 µm and 40 µm from the slag aggregate surface were nearly equal to those at 40 µm and 50 µm from the sand aggregate surface, respectively. Similarly, at a distance of 30 µm from the aggregate surface, the microhardness of the specimens with slag aggregates was 6.9% higher than that of the sand-incorporated mortar. On the other hand, beyond 50 µm from the aggregate surface, microhardness values were nearly identical for both the aggregates. This is attributed to the fact that water inside the slag aggregates was released and contributed to the hydration process near the aggregate surface. In addition to this, the rough surface of the slag aggregate modified the packing of cement particles near the aggregate, which resulted in a denser interfacial transition zone. This phenomenon has been thoroughly discussed in a previous study by the authors [27].

### 3.4. Ultrasonic Pulse Velocity

**Figure 6:**
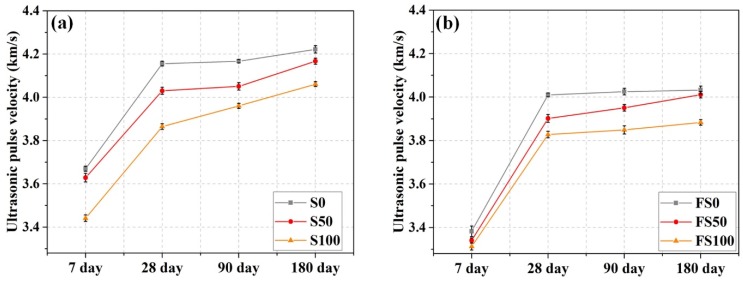
UPV of mortar specimens (**a)** without fiber and (**b**) with fiber.

Ultrasonic pulse velocity was higher for the denser cement composites. Figure 6 shows the results for ultrasonic pulse velocity at 7, 28, 90, and 180 days of curing age for different mortar mixes. The results for specimens without fiber are presented in Figure 6a. It can be seen that UPV decreases with the inclusion of slag aggregate. For S50 and S100, pulse velocity decreased, respectively, 1.1% and 6.2% at 7 days of curing; 3% and 7% at 28 days of curing; 2.8% and 5% at 90 days of curing; and 1.3% and 3.8% at 180 days of curing, in comparison with mortar mix S0. The decrease in UPV is due to the porous structure of the slag aggregate, which increased the overall porosity of the cement composite. It has been established that porosity of the cement matrix causes a reduction in pulse velocity since ultrasonic waves do not propagate quickly in a porous medium [36,37].

Figure 6b illustrates the UPV test results for the mortar specimens with fiber. The addition of fiber further decreased the pulse velocity, irrespective of slag aggregate inclusion. Mortar mixes FS0, FS50, and FS100 showed decreases, respectively, of 7.8%, 7.9%, 3.7% at 7 days of curing; 3.5%, 3.2%, 1% at 28 days of curing; 3.4%, 2.5%, 2.8% at 90 days of curing; and 4.5%, 3.7%, 4.4% at 180 days of curing compared to corresponding mixes without fiber. Similar results were reported in previous studies, with UPV decreasing when PP fibers were added [38,39]. The addition of PP fibers reduced the ultrasonic wave velocity by improving pore inner conductivity [40].

### 3.5. Characteristic Properties in Sulfate Solution

#### 3.5.1. Length and Mass Change

**Figure 7:**
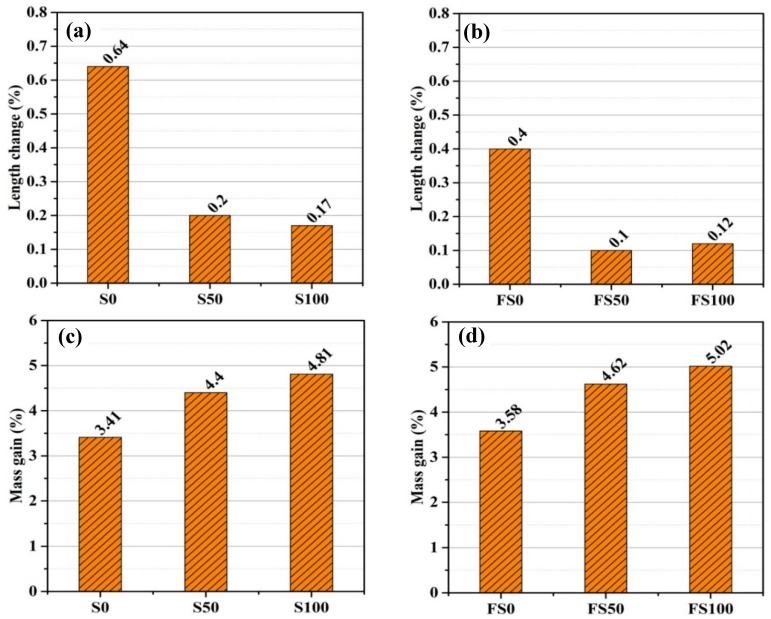
Length change of mortar specimens (**a**) without fiber and (**b**) with fiber; a mass gain of mortar specimens (**c**) without fiber and (**d**) with fiber, exposed to sulfate solution.

Figure 7 shows the changes in mass and length of the mini prisms that were immersed in sodium sulfate solution for a period of 84 days. Mass increased significantly, while length showed only a minor change. For the specimens without fiber, length increases of 0.64%, 0.20%, and 0.17% were observed for S0, S50, and S100, respectively (Figure 7a). On the other hand, mass for mortar mixes S0, S50, and S100 (without fiber) showed increases of 3.41%, 4.40%, and 4.81%, respectively (Figure 7c). For the specimens with fiber, mortar mixes SF0, SF50, and SF100 showed, respectively, an increment of 3.58%, 4.62%, and 5.02% in mass (Figure 7d). On the other hand, the length increase for the specimens with fiber was 0.4%, 0.1%, and 0.12% for FS0, FS50, and FS100, respectively (Figure 7b).

#### 3.5.2. Visual Inspection

**Figure 8:**
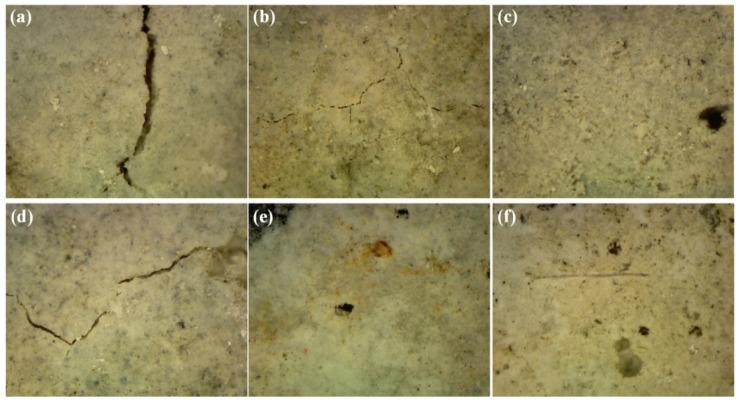
Cracks developed on the surface of (**a**) S0, (**b**) S50, (**c**) S100, (**d**) FS0, (**e**) FS50, and (**f**) FS100 specimens exposed to sulfate solution.

Cracks were observed via microscope on the surface of the specimens (mini prisms) after immersion in sulfate solution, as shown in Figure 8. Wider cracks were detected on the surface of the mortar specimens with only sand as a fine aggregate (Figure 8a) than on those with 50% slag aggregate (Figure 8b). In contrast, no cracks were observed on the surface of mortar specimens with 100% slag as a fine aggregate (Figure 8c). Figure 8d–f present the microscopic images of FS0, FS50, and FS100 specimens, respectively, after exposure to sulfate solution. It can be seen that the cracks that appeared on the surface of fiber-reinforced specimens containing 0% slag aggregate showed narrower cracks in comparison with the respective specimen without fiber. In addition, FS50 and FS100 showed no cracks on the surface after the sulfate attack. It is obvious that fiber reinforcement, along with slag aggregate, intervened in the development of cracks. 

#### 3.5.3. Compressive Strength

Table 2 summarizes the results of compressive strength test employed on mortar specimens immersed in sulfate solution for 180 days. It can be seen that compressive strength significantly increased with immersion time. However, at the immersion age of 180 days, the specimens containing 0% slag aggregate showed a decrease in compressive strength. The results in Table 2 show that mortar mixes S0, S50, and S100 immersed in sulfate solution showed variations, respectively, of 3.3%, 9.0%, 20.1% at 28 days; 2.9%, 5.4%, 8.6% at 90 days; and −2.3%, 13.4%, 4.2% at 180 days, compared to corresponding mixes immersed in water. Binici et al. [41] studied the performance of concrete containing GBFS as fine aggregate after immersion in a sodium sulfate solution. GBFS was used at a replacement percentage of 5%, 10%, and 15% by weight of sand, and the specimens were kept in 5% Na_2_SO_4_ solution for 180 days. The results of their study confirmed that the sulfate resistance of the concrete specimens increased with the addition of GBFS aggregates. The least mass loss and highest compressive strength were obtained for concrete specimens with 15% GBFS. Results of a study conducted by Lee et al. [42] indicate that mortar specimens containing 0% and 25% recycled fine aggregate presented an increase in compressive strength when exposed to sodium sulfate solution up to 180 days. It has been well established that incorporation of slag aggregate imparts a porous microstructure in cement composites [5,28,32]. Later on, secondary ettringite crystals form within the pores, resulting in a densified microstructure [3]. This may be the reason for the weight gain and enhanced compressive strength of mortar specimens containing slag aggregates after exposure to sulfate solution. 

The mortar specimens SF0, SF50, and SF100 immersed in sulfate solution presented a variation in compressive strength, respectively, of 9.9%, 10.2%, 18.8% at 28 days; 2.5%, 7.4%, 6.1% at 90 days; and −1.0%, 5.1%, 3.5% at 180 days, compared with the corresponding specimens immersed in water. The compressive strength enhancement in sulfate solution may be due to the formation of gypsum or ettringite in the empty voids of the mortar mixes, making the microstructure denser, as it has been indicated that fiber inclusion increases the porosity of the cement composites [14].

### 3.6. SEM and XRD Analysis

**Figure 9:**
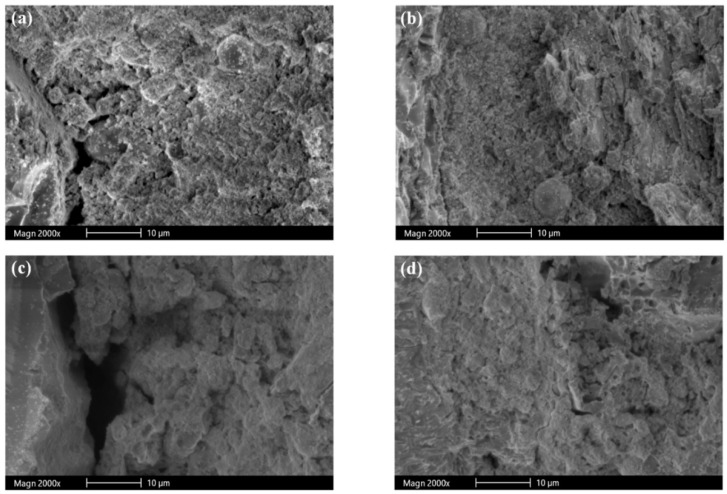
SEM micrographs of (**a**) S0 and (**b**) S100 specimens stored in water; (**c**) S0 and (**d**) S100 specimens exposed to sulfate solution.

Figure 9 portrays micrographs of SEM analysis conducted on the mortar specimens exposed to water and sulfate solutions. Figure 9a,b present the SEM micrographs of S0 and S100 specimens stored in water for 28 days. It is clear that the slag mortars showed a denser microstructure than the sand mortars. In particular, the aggregate-paste interface for the slag-containing mortars was dense, while that of the sand mortars showed a porous interface between aggregate and cement paste. This is mainly attributed to the porous and rough surface of the slag aggregates, which provided better packing of the cementitious particles, and released water absorbed during hydration, thus enhancing the microstructure near the aggregate surface. Figure 9c,d display the SEM micrographs of sand (S0) and slag (S100) mortar specimens (mini prisms) exposed to sulfate solution for 84 days, respectively. It is evident from the figures that the paste was detached from the sand aggregate, while the microstructure of the slag-paste interface was dense. This is due to the fact that the slag aggregates absorbed the expansion stresses caused by sulfate attack. This behavior of the slag mortars in sulfate solution resulted in higher compressive strength in comparison to the sand-containing mortars. 

**Figure 10:**
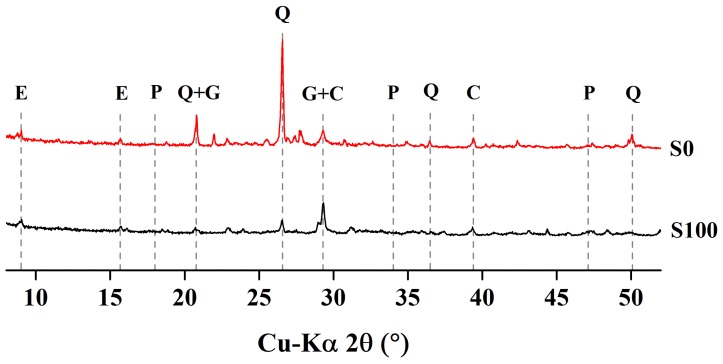
XRD patterns of mortar specimens stored in water for 28 days.

**Figure 11:**
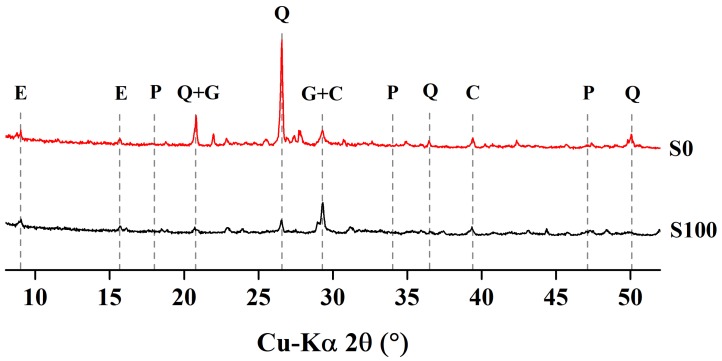
XRD patterns of mortar specimens exposed to sulfate solution for 84 days.

XRD analysis was used to detect the changes in crystalline phases of the cement mortar specimens containing natural sand, as well as those containing slag as fine aggregate. Both specimen types were exposed to water and to sulfate solution (mini prisms were exposed to sulfate solution). Figure 10 shows the XRD patterns for mortar specimens cured in water, while Figure 11 presents the XRD patterns for those exposed to sulfate solution for 84 days. The water-cured specimens presented major peaks of portlandite and quartz, along with several minor peaks for ettringite and calcium aluminate monosulfate (Afm). For specimens immersed in sulfate solution, the portlandite peak vanished, while the relative peaks for gypsum and ettringite were increased. This is mainly due to the reaction of portlandite with sulfate ions, forming ettringite and gypsum minerals. The specimens cured in water for 28 days showed major quartz peaks. The relative peaks for quartz were smaller in the slag aggregate specimens than in the sand aggregate specimens. The portlandite peaks in the slag aggregate specimens were also smaller than in the sand aggregate specimens. This may be due to the partial carbonization of Ca(OH)_2_, which resulted in an emerged peak of calcite at 29.4°. On the other hand, slag aggregate specimens subjected to sulfate solution showed no peaks for portlandite, and showed enhanced peaks for ettringite and gypsum, confirming the sulfate attack. 

## 4. Conclusions

This study utilized fly ash and silica fume as cement replacement materials. In addition, air-cooled blast furnace slag aggregates were used to replace fine aggregate 100% by weight. A set of mortar mixes with a PP fiber dosage of 0.25% by cement weight was also prepared. The mortar specimens were examined in terms of various parameters. Based on the results of this study, the following conclusions can be made.

1. The inclusion of slag aggregate increases the flow of mortar mixes, while the addition of fiber reduces the flow;
2. The incorporation of 50% slag aggregate results in enhanced compressive, flexure, and splitting tensile strength. Strength decreases with a replacement level beyond 50%. The strength values for fiber-reinforced specimens are higher than those for specimens without fiber;
3. Slag aggregates provide better packing of cementitious particles near the aggregate surface than sand aggregates provide. Consequently, a dense microstructure in the near aggregate surface for slag specimens is observed;
4. The addition of slag aggregate increases the porosity of mortar specimens. This results in lower UPV values;
5. Compared to mortar specimens containing only sand as a fine aggregate, slag-incorporated mortar specimens present enhanced performance under sulfate attack. The formation of gypsum and ettringite in the empty voids of slag mortars densifies the microstructure, which results in increased compressive strength.

## 5. Further Research

Future research will focus on the influence of slag replacement, with small variations in the replacement percentage, up to 50%. The exposure to sulfate solution densified the microstructure of the mortar specimens. Therefore, future research will also be conducted to investigate the carbonation and chloride penetration of the mortar specimens after exposure to sulfate solution. 

## Figures and Tables

**Table 1:** Chemical and physical properties of ordinary Portland cement (OPC), fly ash (FA), and silica fume (SF).

Composition	Weight (%)		
	OPC	FA	SF
SiO_2_	20.8	56.6	88.7
Al_2_O_3_	6.3	27.1	1.8
Fe_2_O_3_	3.2	4.4	1.8
CaO	62	3.8	1.5
MgO	3.3	0.8	0.8
SO_3_	2.2	0.2	0.1
Physical properties	-	-	-
Specific gravity	3.15	2.23	2.2
Specific surface area (cm²/g)	3200	3750	20,000
Loss on Ignition (%)	1.3	4.4	1.1

**Table 2:** Compressive strength of the mortar specimens immersed in water and sulfate solution (SD values in parenthesis).

Mortar Mix	Compressive Strength (MPa)					
	28 Days		90 Days		180 Days	
	Water Immersed	Sulfate Immersed	Water Immersed	Sulfate Immersed	Water Immersed	Sulfate Immersed
S0	36.3 (3.1)	37.5 (2.6)	47.8 (3.3)	49.2 (2.1)	50.3 (1.8)	49.1 (1.3)
S50	44.1 (2.4)	48.1 (2.0)	57.6 (1.7)	60.7 (1.2)	58.8 (1.5)	66.7 (1.6)
S100	28.8 (1.5)	34.6 (1.1)	46.6 (1.8)	50.6 (2.4)	47.8 (2.1)	49.8 (1.4)
FS0	35.3 (1.1)	38.8 (1.7)	51.0 (2.1)	52.3 (3.2)	52.0 (1.6)	51.5 (2.6)
FS50	50.3 (1.7)	55.4 (1.5)	60.1 (2.0)	64.5 (1.4)	60.1 (2.2)	63.1 (1.8)
FS100	31.4 (2.0)	37.3 (1.9)	47.8 (1.5)	50.7 (1.9)	48.6 (2.3)	50.3 (2.0)
